# Long-term outcomes and recurrence pattern of 18F-FDG PET-CT complete metabolic response in the first-line treatment of metastatic colorectal cancer: a lesion-based and patient-based analysis

**DOI:** 10.1186/s12885-018-4687-9

**Published:** 2018-07-31

**Authors:** Keith W. H. Chiu, Ka-On Lam, H. An, Gavin T. C. Cheung, Johnny K. S. Lau, Tim-Shing Choy, Victor H. F. Lee

**Affiliations:** 1 Department of Diagnostic Radiology, LKS Faculty of Medicine, The University of Hong Kong, 102 Pokfulam Raod, Hong Kong, China; 2 Department of Clinical Oncology, LKS Faculty of Medicine, The University of Hong Kong, 1/F Professorial Block, 102 Pokfulam Raod, Hong Kong, China; 3 Clinical Oncology Center, The University of Hong Kong-Shenzhen Hospital, 102 Pokfulam Raod, Hong Kong, China; 4 Department of Clinical Oncology, Queen Elizabeth Hospital, 30 Gascoigne Raod, Hong Kong, China; 5 Department of Clinical Oncology, Queen Mary Hospital, 1/F Professorial Block, 102 Pokfulam Raod, Hong Kong, China

**Keywords:** Metastatic colorectal cancer, 18F-FDG PET, Complete metabolic response, Systemic therapy, Palliative

## Abstract

**Background:**

18F-FDG PET-CT is commonly used to monitor treatment response in patients with metastatic colorectal cancer (mCRC). With improvement in systemic therapy, complete metabolic response (CMR) is increasingly encountered but its clinical significance is undefined. The study examined the long-term outcomes and recurrence patterns in these patients.

**Methods:**

Consecutive patients with mCRC who achieved CMR on PET-CT during first-line systemic therapy were retrospectively analysed. Measurable and non-measurable lesions identified on baseline PET-CT were compared with Response Criteria in Solid Tumors (RECIST) on CT on a per-lesion basis. Progression free (PFS) and Overall Survival (OS) were compared with clinical parameters and treatment characteristics on a per-patient basis.

**Results:**

Between 2008 and 2011, 40 patients with 192 serial PET-CT scans were eligible for analysis involving 44 measurable and 38 non-measurable lesions in 59 metastatic sites. On a per-lesion basis, 46% also achieved Complete Response (CR) on RECIST criteria and sustained CMR was more frequent in these lesions (OR 1.727, *p* = 0.0031). Progressive metabolic disease (PMD) was seen in 12% of lesions, with liver metastasis the most common. Receiver operating characteristics (ROC) curve analysis revealed the optimal value of SUVmax for predicting PMD of a lesion was 4.4 (AUC 0.734, *p* = 0.004). On a per-patient basis, 14 patients achieved sustained CMR and their outcomes were better than those with PMD (median OS not reached vs 37.7 months *p* = 0.0001). No statistical difference was seen in OS between patients who achieved PR or CR (median OS 51.4 vs 44.2 months *p* = 0.766).

**Conclusion:**

Our results provided additional information of long-term outcomes and recurrence patterns of patients with mCRC after achieving CMR. They had improved survival and sustained CMR using systemic therapy alone is possible. Discordance between morphological and metabolic response was consistent with reported literature but in the presence of CMR the two groups had comparable outcomes.

## Background

Colorectal cancer is the third commonest cancer worldwide with nearly 1.4 million new cases diagnosed annually. [1] Nearly 20% of patients are diagnosed late and curative surgery is not possible due to extensive metastatic disease [2]. For this group of patients, palliative chemotherapy with or without biological agents are recommended. [3, 4] As the aims of treatment in these patients are to prolong survival and improve quality of life, it is prudent to identify responders from non-responders to avoid futile treatments and excessive toxicities.

Currently, contrast enhanced computed tomography (CT) is the imaging modality of choice and the Response Evaluation Criteria In Solid Tumors (RECIST) is commonly applied in assessing treatment response. [5] However, as RECIST is based on anatomical changes in size of the tumor, its correlation with morphological alterations and patient outcomes, particularly in the era of novel combination chemotherapy and biological agents are unclear. [6]

^18^F-2-fluoro-2-deoxy-D-glucose (^18^F-FDG) positron emission tomography-computed tomography (PET-CT) is an imaging modality that measures and quantifies metabolic avidity in cancer cells, thus serving as a proxy for the underlying cellular activity and viability. [7] It is widely used in the diagnosis, prognostication and treatment response in many cancers although its use in colorectal cancer remains controversial. [8] Previous studies have shown its sensitivity in detecting treatment response and those with metabolic response (mR) might have improved clinical outcome. [9–11]

Complete metabolic response (CMR) is a distinct clinical entity and it is increasingly encountered with the advent of systemic therapy in patients with metastatic colorectal cancer (mCRC). However, little is known whether achieving CMR, with or without RECIST response, confers survival advantage or merely represents a transient quiescence in FDG-uptake with limited clinical impact. To study the clinical significance of CMR in mCRC, we performed a retrospective analysis using both lesion-based and patient-based approaches on consecutive patients in a prospectively-maintained database.

## Methods

### Patients

Institutional review board approval with waiver of informed consent of individual patients was obtained for this retrospective non-interventional study (HKU/HA HKW IRB: UW 15–315). Adult patients with mCRC referred to the Department of Clinical Oncology, Queen Mary Hospital, Hong Kong for management from 2008 to 2011 were reviewed in a prospectively-maintained departmental database. Consecutive patients with both baseline and serial ^18^F-FDG PET-CT who achieved CMR using fluoropyrimidine-based systemic therapy in the first-line setting were included for further analysis. Patients who achieved CMR from surgery were excluded. Patient demographics and treatment characteristics were recorded.

### ^18^F-FDG PET-CT examinations and image analysis

Whole-body 18F FDG PET-CT was performed using a GE Discovery 610 PET-CT (GE Healthcare, Milwaukee, WI, field of view 50 cm; pixel size, 3.91 mm; spiral CT pitch, 0.984; gantry rotation speed, 0.5 s) using a standard protocol. After at least 6 h of fasting (and with serum glucose level < 10 mmol/l), an intravenous injection of 222-370 MBq (4.8 MBq/kg) of weight adjusted 18F FDG was administered. Uptake time was 1 h. Whole body emission PET scan was obtained with six bed positions of 2 min acquisition time in each bed position. Attenuation-corrected PET images with CT data were reconstructed with an ordered-subset expectation maximization iterative reconstruction algorithm (14 subsets and two iteration) and fused with CT images (Advanced Workstation 4.7, GE Healthcare, Milwaukee, WI). The CT imaging parameters were 120 kV, auto mA, pitch of 0.98 and rotation time of 0.5 s. Iodinated contrast (Iopamidol-370, Bracco Diagnostics Inc., Italy) at 1 mg/kg was administered intravenously at a rate no less than 3 ml/s via a peripheral cannula. Portovenous phase images at 70 s was obtained after intravenous contrast administration. Most (72%) of ^18^F-FDG PET-CT images were acquired at the Department of Diagnostic Radiology, The University of Hong Kong using a GE Discovery 610 PET-CT (GE Healthcare, Milwaukee, WI) and remaining scans were performed using a GE Discovery 690 PET-CT (GE Healthcare, Milwaukee, WI) from another institute. All PET-CT were performed using the same protocol and all patients were scanned using the same scanner throughout their follow-up.

Baseline ^18^F-FDG PET-CT were performed before the commencement of systemic therapy and follow-up PET-CT were performed 3-monthly until progressive disease or as per clinical need after sustained CMR. All PET-CT images were reviewed by a board-certified radiologist and a radiation oncologist both with experience in PET-CT by consensus blinded to both medical records and treatment outcomes. The metabolic response (mR) process comprised of four phases: identifying indexed lesions on baseline PET-CT; assessing the mR of each target lesion; categorizing the mR distribution; and dichotomizing the overall mR. All lesions were reviewed for their metabolic activity against background liver on visual inspection. Criteria for indexed lesions and response evaluation on PET-CT were based on PERCIST criteria version 1.0. [12]

Lesion-based and patient-based analyses were performed on the PET component of all follow-up PET-CT. Response were characterized into complete metabolic response (CMR), partial metabolic response (PMR), stable metabolic disease (SMD) and progressive metabolic disease (PMD) based on comparison with the immediate prior PET-CT.

CMR was defined as complete resolution of FDG uptake in both target and non-target lesions, with FDG uptake less than the mean SUV normalized to lean body mass (SUL) of the liver and indistinguishable from the surrounding background and no new FDG-avid lesions in a pattern typical of cancer appearance. Sustained CMR was defined as progression-free status after achieving CMR with no further PMD for at least 24 consecutive months. For disease progression after CMR, it was defined as lesions showing an increase of greater than or equal to 30% and an increased at least 0.8 SUL units of ^18^F-FDG uptake in a target lesion or development of one or more new lesions in a pattern typical of metastatic spread of the cancer. A lesion was considered new when it was first visualized, even if retrospective reviewed deemed to have been faintly present earlier. [12]

Criteria for indexed lesions and response evaluation on CT was assessed per RECIST criteria version 1.1. [5] Lesion-based and patient-based analyses were performed on the CT component of all follow-up PET-CT. Response was calculated as the change in the longest diameter between the baseline and follow-up CT and measurable lesions were classified as complete response (CR), partial response (PR), stable disease (SD) and progressive disease (PD). Non-measurable disease was noted as either present or absent on follow-up scans.

### Statistical analysis

Statistical analysis was performed using SPSS version 23 (IBM Corp., NY, US). Kaplan-Meier method was used for progression-free survival (PFS) and overall survival (OS) analysis with log-rank test for *P*-value calculation and Cox-regression analyses for hazard ratio (HR) and confidence interval (CI) calculations. Univariate logistic regression models were used to calculate odds ratios (OR) and *p*-values. PFS for RECIST criteria was defined as the period between the commencement of systemic therapy and when PD was identified and PFS for PERCIST criteria was defined as the period between commencement of systemic therapy and when PMD was identified. OS was defined as time from commencement of systemic therapy until all cause of death. A receiver operating characteristic (ROC) analysis was carried out to define the optimal cut-off of SUVmax of metastatic lesions on staging PET-CT in predicting PMD and overall survival.

## Results

### Demographics

Between 2008 and 2011, 1007 patients were referred to our department for management of CRC. Three-hundred and fifty-six patients (35%) received at least one course of systemic therapy for metastatic disease. Amongst them, 202 patients (20%) had undergone baseline and serial PET/CT for treatment response. Forty patients achieved CMR with chemotherapy either alone or with biological agents. The median follow-up time was 47 months (range, 7–109 months).

The median age was 60 years (range, 35–80 years). Twenty-four patients had adenocarcinoma in the colon and 16 in the rectum. Using splenic flexure (Griffith’s point) as division, 9 were in the right colon and 31 in the left colon or rectum. Thirty-seven patients (93%) had primary tumor resected before systemic therapy. All patients were evaluated at their first-line treatment. Patient demographics and treatment characteristics were summarized in Table 1.

**Table 1:** Patient demographics and treatment characteristics (*n* = 40). Mann-Whitney U test was used for continuous variables and Chi-Square test was used for categorical variables

	All CMR	Sustained CMR	PMD	*P* value
Median Age (years)	60 (35–80)	62 (35–74)	59 (41–80)	0.408
Sex (Male:Female)	24:16	5:9	19:7	0.021
ECOG status				
• 0	3	3	0	
• 1	35	11	24	0.013
• 2	2	0	2	
Primary site				
• Right colon	9	5	4	0.149
• Left colon	31	9	22	
T staging				
• T3	16	7	9	0.266
• T4	18	6	12	
• Tx	6	1	5	
N staging				
• N0	2	1	1	
• N1	15	6	9	0.432
• N2	18	6	12	
• Nx	5	1	4	
KRAS status				
• Wild type	29	11	18	0.355
• Mutation	8	1	7	
• Unknown	3	2	1	
Metastatic Site				
• Liver	17	3	14	0.049
• Lung	7	3	4	0.416
• Lymph node	20	8	12	0.520
• Peritoneal	11	4	7	0.914
• Others	4	2	2	0.520
Chemotherapy regime				
• XELOX	29	9	20	
• FOLFOX	5	3	2	
• XELIRI	2	1	1	0.989
• FOLFIRI	2	1	1	
• XELODA	2	0	2	
Biological agent				
• None	16	9	7	
• Bevacizumab	8	1	7	0.021
• Cetuximab	16	4	12	
• Median number of chemotherapy cycle to achieve complete metabolic response (CMR)	4 (3–12)	5 (4–12)	4 (3–10)	0.234
• Median number of chemotherapy cycles received after CMR was achieved	3 (0–8)	3 (0–8)	3 (0–8)	0.980

Baseline PET-CT was acquired in all patients with 3 performed prior to resection of the primary tumor. They underwent a total of 192 PET-CT with a median of 4 scans per patients (range, 2–13 scans). The median number of cycles of systemic therapy before CMR was 4 (range 3–12) and median total number of cycles of first-line systemic therapy was 8 (range 3–12). At the time of analysis, 26 patients had subsequent PMD and all died from disease-related causes. The median PFS and OS were 15.6 (95% CI 1.2–30.7) months and 44.6 (95% CI 33.3–56.0) months, respectively. Two patients achieved further CMR with second line treatment after first PMD. Amongst the 16 who achieved sustained CMR (CMR lasting for > 24 months), two developed PMD 25.5 and 28.5 months and a further two patients died of unrelated causes 36.1 and 47.1 months after achieving CMR. Those who achieved sustained CMR in the first line treatment comprised significantly more female patients, patients with better performance status but less use of biological agents (Table 1).

### Lesion-based analysis of treatment response

Baseline PET-CT identified 52 measurable lesions and a further 41 non-measurable lesions at a total of 65 metastatic sites. Four liver metastases and a single nodal disease in 3 patients were excluded for analysis as their baseline PET-CTs were acquired prior to surgical resection of primary tumor and these metastatic diseases were removed concurrently with the primary tumor at surgery. In all 3 cases, other metastatic diseases were present and removal of these lesions did not result in either CMR or CR for the patients. Three liver metastases, two peritoneal diseases and a lung metastasis from 4 patients were surgically removed after CMR was achieved and they were also excluded from lesion-based analysis. In total, 44 measurable and 38 non-measurable lesions from 59 metastatic sites were included. The frequency of specific metastases at diagnosis is summarized in Table 2.

**Table 2:** Distribution of measurable and non-measurable lesions on baseline PET-CT

	Metastatic sites	Measurable lesions	Non-measurable lesions
Total No	59	44	38
Liver	17	27	5
Lung	6	1	5
Lymph Nodes	20	7	18
Peritoneal	11	7	7
Others	5	2	3

**Fig. 1:**
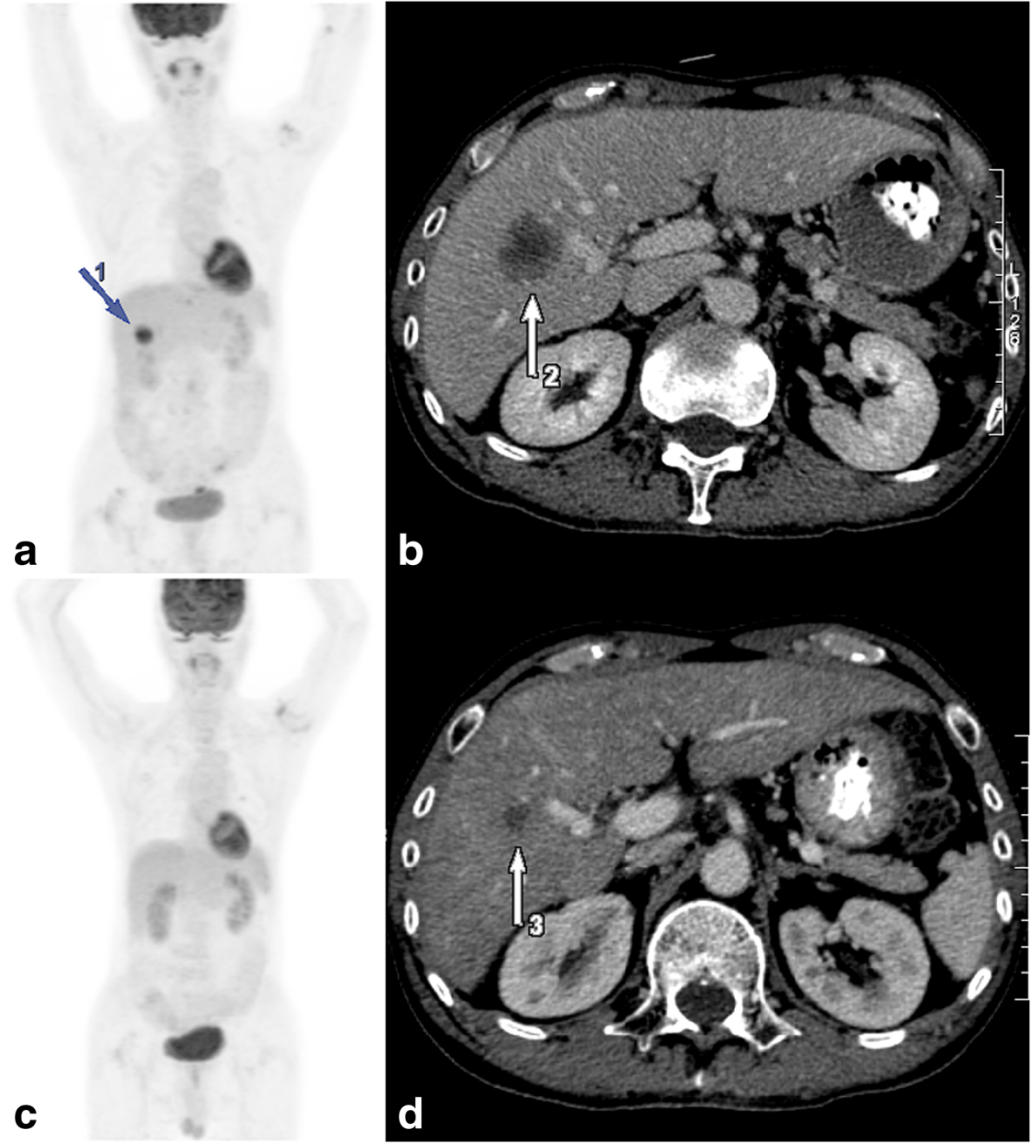
Discordance between PERCIST and RECIST criteria in a 66-year-old male patient with kras wild-type T4N1M1 recto-sigmoid adenocarcinoma. Top row: PET and CT images performed at baseline after surgical resection of the primary tumor. **a** Maximum-intensity-projection (MIP) image shows a hypermetabolic mass in segment V/VIII of the liver (blue arrow 1). **b** Corresponding contrast enhanced CT image confirming the presence of a liver metastasis (white arrow 2). Bottom row: PET and CT images after 10 cycles of XELOX and cetuximab. **c** MIP image shows CMR compared to (**d**) CT image demonstrates a residual lesion (white arrow 3). The patient was considered to have partial response to treatment by RECIST criteria. The patient was considered to have progressive metabolic disease after 5 months of complete metabolic response

**Fig. 2:**
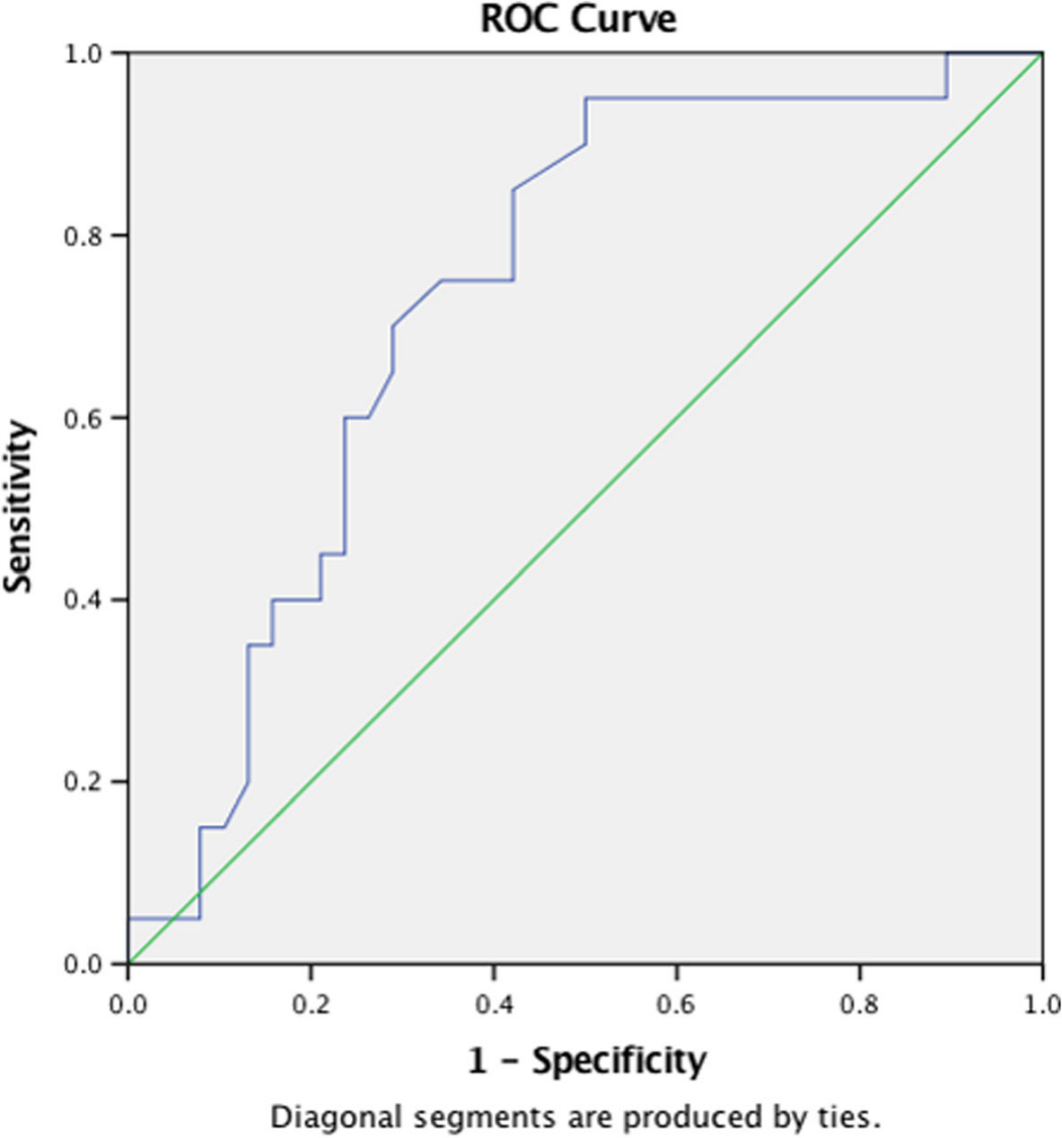
ROC curve of using SUVmax to predict disease progression of individual lesion. A SUV max of 4.4 has a 70% sensitivity and 71% specificity of predicting progressive disease of the individual lesion. The ROC AUC is 0.734 (95% CI 0.602–0.865, *p* = 0.004)

Of the 82 lesions included for analysis, 46% (38/82) achieved CR as per RECIST criteria. Sustained CMR was significantly more frequent in lesions that achieved CR on corresponding CT (OR 1.727, 95%CI 1.206–2.627 *p* = 0.0031) (Fig. 1). Univariate analysis has shown that liver metastases with partial response are significantly more likely to develop disease progression compared with liver lesions with CR on CT (OR 7.333 95%CI 1.329–29.84; *p* = 0.0155). The size of the measurable lesions on CT did not predict whether PMD would occur after achieving CMR (OR1.206 95%CI 0.839–1.734 *p* = 0.311). On the other hand, SUVmax of the lesions were shown to be predictive of subsequent PMD. The ROC area under curve (AUC) of available SUVmax of the recorded lesions (58/82) was 0.734 (SE 0.067 95% CI, 0.602–0.865; *p* = 0.004) in predicting subsequent PMD with a sensitivity of 70.0% (95% CI 46.9 to 86.7%) and specificity of 71.1% (95% CI 55.2 to 83.0%) for lesions with SUVmax of 4.4 or above (Fig. 2).

### Patient-based analysis of treatment response

Twenty patients had metastatic disease involving a single site, although they had multiple lesions within the single site. Twenty-two patients (55%) achieved both CR and CMR. Of the remaining, 16 (40%) had PR and 2 (5%) had SD based on RECIST criteria. In contrast to lesion-based analysis, no significant correlation of sustained CMR and CR was demonstrated per patient-based analysis (OR1.800 95%CI 0.4751–6.683 *p* = 0.5103). No statistical difference was found in median PFS and OS between those with CR and PR/SD (25.5 vs 14.4 months, HR 1.544 95% CI 0.714–3.338; *p* = 0.270 and 44.2 vs 51.4 months, HR 1.12 95% CI: 0.530–2.370; *p* = 0.766, respectively).

**Fig. 3:**
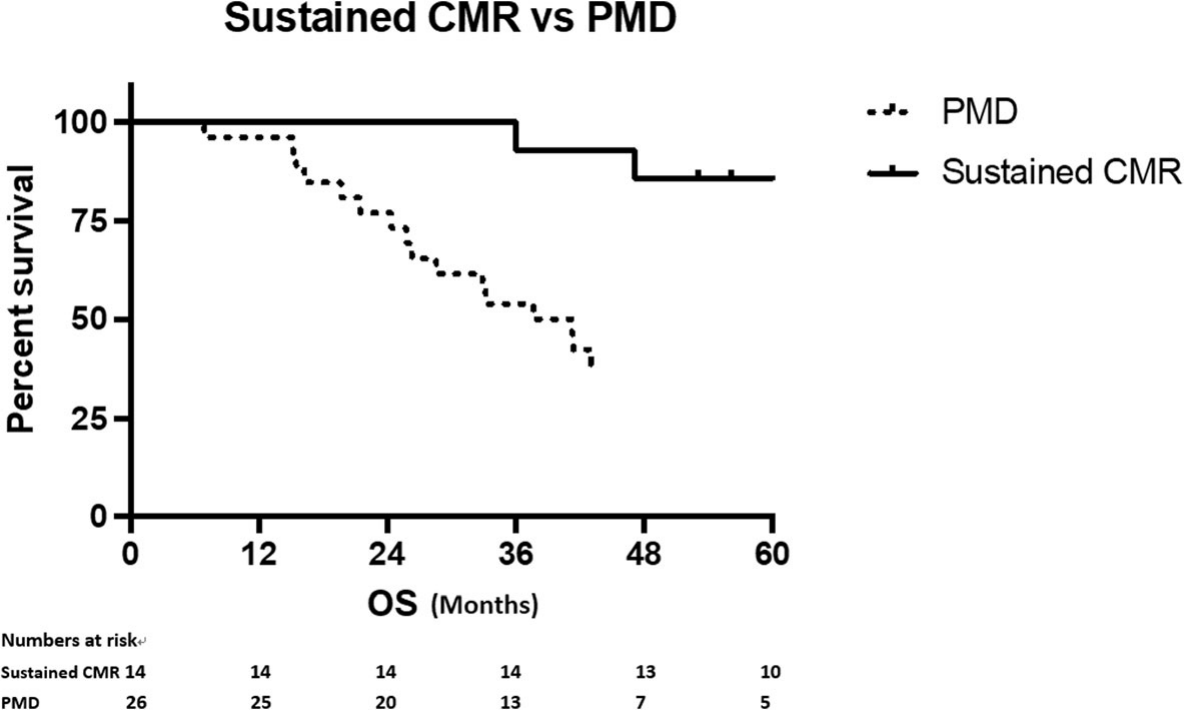
Kaplan-Meier survival plots according to whether patient achieved sustained CMR. Median OS for patients who had sustained CMR were significantly longer than those who had subsequent PMD (Not reached vs 37.7 months, HR 5.329 95%CI 2.481–11.45, *p* < 0.001)

In the 26 patients that subsequently had PMD, 6 were in previously known locations only, 6 in both known and new locations and 14 were in new locations only. Eleven patients (42.3%) had lesions in multiple sites at confirmation of PMD. The most common site for metastases when subsequent PMD was detected was the liver (10/41 sites, 7/17 previously indexed locations). The most common locations for new lesions were nodal and peritoneal diseases (both 26%). Overall survival was significantly longer in patients who achieved sustained CMR (median OS not reached vs 37.7 months, HR 5.329 95% CI 2.481–11.45; *p* < 0.001). (Fig. 3).

### Correlation with CEA

Serum CEA levels were elevated (> 5 ng/ml) in 21/40 patients at diagnosis. Patients with normal baseline serum CEA were more likely to have sustained CMR (OR 4.722, 95%CI 1.163–16.1; *p* = 0.044). When CMR was achieved, serum CEA was normal in 34/40 patients, with normalization of CEA levels in 15/21 patients. Univariate analysis has shown normal CEA levels at diagnosis had statistically superior survival outcomes. (Table 3).

**Table 3:** Baseline characteristics and association with survival outcomes

Characteristics	*N*	%	Median PFS (months)	HR (95% CI), *p* value	Median OS (months)	HR (95% CI), *p* value
Overall cohort	40	100	15.6		44.6	
Sex						
• Male	24	60.0	14.3	0.473 (0.205–1.092)	43.7	0.591 (0.267–1.308)
• Female	16	40.0	45.3	*p* = 0.080	51.3	*p* = 0.194
Age						
• < 60	20	50.0%	15.4	0.691 (0.317–1.509)	43.1	0.569 (0.264–1.223)
• > 60	20	50.0%	16.0	*p* = 0.354	63.1	*p* = 0.149
T stage						
• T3	16	40.0%	14.4	0.975 (0.413–2.299)	51.3	1.363 (0.581–3.194)
• T4	18	45.0%	25.5	*p* = 0.954	44.6	*p* = 0.477
N stage						
• N1	16	40.0%	13.3	1.108 (0.564–2.175)	43.7	0.978 (0.516–1.852)
• N2	17	42.5%	16.0	*p* = 0.767	61.7	*p* = 0.945
Serum CEA level at diagnosis						
• Normal	19	47.5%	48.7	2.879 (1.269–6.534)	74.2	2.355 (1.081–5.133)
• Raised	21	52.5%	12.1	*p* = 0.011	41.4	*p* = 0.031
Metastatic sites						
• Single	22	55.0%	16.1	1.090 (0.504–2.361)	44.2	0.869 (0.406–1.859)
• Multiple	18	45.0%	14.4	*p* = 0.826	51.4	*p* = 0.717

Patients on biological therapy were statistically less likely to achieve sustained CMR compared with those on chemotherapy alone on univariate analysis (20.8% vs 56.3%, OR 0.194 95% CI 0.046–0.790; *p* = 0.023).

## Discussion

Metabolic response to systemic therapy is a well-recognized prognostic marker for improved survival but the clinical impact of achieving CMR in patients with mCRC remains to be elucidated [11]. To our knowledge, this represented the largest cohort of CMR reported in the literature with uniform imaging technique and mature follow-up data (median follow-up time of 47 months) that highlighted the long-term outcomes and recurrence patterns. The PFS and OS of 15.6 and 44.6 months, respectively, compared favorably to contemporary literature in the biomarker-selected population. [13–15]

Despite significant improvement in survival, there remains a discrepancy between achieving CMR on PET-CT and complete eradication of disease. Normalization of ^18^F-FDG uptake in metastases after chemotherapy has been described as CMR but the long term outcomes and recurrence pattern of these lesions remain unclear. [16–19] Our results have shown that sustained CMR is possible in 40% of CMR patients and those who achieved sustained CMR have significantly prolonged survival. The outcomes did not appear to be the result of post-CMR or maintenance therapy as the median number of cycles of systemic therapy were similar for patients with sustained CMR and PMD (Table 1).

In accordance with our study, a previous study has also shown that pre-treatment SUVmax of the main metastatic lesion was associated with OS [20]. Furthermore, we have shown that high SUVmax (> 4.4) of individual lesion was predictive of subsequent PMD while sustained CMR was more frequent in individual lesions with RECIST CR on a lesion-based analysis. Thus, initial SUVmax and the corresponding RECIST response of individual lesion may guide decisions for radical local therapy e.g. resection or stereotactic body radiotherapy, on a lesion-by-lesion basis after achieving CMR with systemic therapy. Further research is required to decipher the relationship between tumor metabolism and sensitivity to systemic treatment, taking into account of other established PET parameters not analyzed in this study such as SUVmean, metabolic tumor volume and total glycolytic volume. [21]

In our cohort, there was considerable discordance between PET-based and CT-based response evaluation with 45% of patients considered PR/SD only on CT. This was consistent with previous studies by Monteil et al. and Skougaard et al. whereby better overall metabolic responses on PET-CT were seen than best overall response on CT. [22, 23] We have analyzed the outcomes of these two groups and shown no statistical difference but the small number of patients did not allow a definitive conclusion. Nevertheless, this suggested that PET-CT was complementary, if not superior, to CT-based analysis for identifying a subgroup of patients that may benefit substantially from systemic treatment alone. A caveat to this finding was that, upon lesion-based analysis, metastasis which only achieved PR on CT are statistically more likely to have PMD. PMD on previously indexed locations implied residual tumor although they did not affect OS in our cohort as 31/41 (75.6%) of lesions identified on PET-CT at the time of subsequent PMD per patient-based analysis were new lesions. It was beyond the scope of this retrospective study to address whether early intervention to those RECIST PR lesions in the presence of CMR would effectively prevent subsequent systemic progression. Furthermore, our results supported the current understanding of mCRC as a systemic disease and aggressive local therapy in the presence of initial widespread disease have to be highly-selective despite CMR, especially in the presence of high initial SUVmax and RECIST non-CR of individual lesion. On the other hand, durable disease control was more likely in those lesions with low baseline SUVmax on PET-CT and RECIST CR after systemic therapy alone, and they might be spared of more aggressive therapy.

Imaging biomarkers aside, we have also demonstrated the prognostic value of serum CEA levels. Pre-operative CEA has previously been shown to be an independent prognostic factor for outcomes [24, 25] and in our cohort, patients with normal serum CEA levels at diagnosis have significantly longer PFS, OS and were more likely to achieve sustained CMR compared to those with a raised serum CEA. Furthermore, normalization of CEA was seen in the majority of patients when CMR was achieved and raised again in those who had subsequent PMD. This added credence to its use for comprehensive treatment response assessment and detection of recurrence in those patients who have already achieved CMR and might only require a less intensive schedule for PET/CT scan. Radiological imaging could be better scheduled when a rising CEA trend is established thus reducing ionizing radiation to the patients and costs to the healthcare system.

There were known limitations intrinsic to a retrospective study, however, due to unpredictability and relatively infrequent occurrence of CMR in mCRC prospective recruitment or randomization of post-CMR treatment of these patients is not feasible.

To minimize selection bias, only patients treated with fluoropyrimidine-based systemic therapy in the first-line setting were included, although some factors that have shown prognostic values such as serum CA 19–9 or LDH level were not mandatory in our prospectively-maintained database. [26–28] Nevertheless, these factors were less relevant to the current study as it was not the intention to derive predictive factors for whom CMR could be achieved, rather the key message was to describe the natural history of patients and lesions with CMR and thus their potential treatment implications. Due to the small size of our cohort as a result of infrequent occurrence of CMR, multivariate analysis was not deemed feasible and that subtle correlations between potential prognostic factors and outcomes may not be demonstrated. Follow-up schedule and arrangement of PET/CT in the real-world setting might introduce bias to the estimation of time-to-event endpoints. The unexpected finding that patients on biological therapy were less likely to achieve sustained CMR may be due to selection bias in this small cohort of patients. Variation in tumor loads and difference in tumor biology for those who responded well to chemotherapy alone vs those who required additional biological therapy might be the underlying reasons.

Despite all these, the uniform assessment methodology applied and the evaluation of only patients with CMR should have improved inter- and intra-observer variability in interpreting the PET reports. Although previous study has shown significant variability in quantifying PET parameters, this was thought to be due to differences in method of attenuation correction and variation in imaging protocol [29]. As all patients were scanned using the same protocol and both scanners employed the same attenuation correction methods, we believe the threshold SUV max value calculated in predicting subsequent PMD is reproducible. The mature results so generated with long-term follow-up of 47 months was also reflective of real-world clinical practice. In summary, our results provided additional information on the long-term outcomes and pattern of recurrence in this distinct subgroup of patients with potential treatment implications.

## Conclusion

Our study showed that patients who achieved CMR on ^18^F-FDG PET-CT have improved clinical outcomes. Although many of them subsequently develop PMD, sustained CMR with systemic therapy was achievable especially with low baseline SUVmax of individual lesion, normal baseline serum CEA as well as RECIST CR at the time of CMR. Discordance was seen between morphological and metabolic treatment response but the two groups had comparable outcomes and we believe PET/CT, especially in those in which aggressive local therapy is contemplated, has a complementary role to cross-sectional imaging in prognostication, treatment monitoring and planning.

## Abbreviations

18F-FDG: 18F-2-fluoro-2-deoxy-D-glucose
CI: Confidence interval
CMR: Complete metabolic response
CR: Complete response
CT: Computed tomography
HR: Hazard ratio
mCRC: Metastatic colorectal cancer
mR: metabolic response
OR: Odds ratios
OS: Overall survival
PD: Progressive disease
PERCIST: Positron Emission Tomography (PET) response criteria in solid tumors
PET-CT: Positron emission tomography-computed tomography
PFS: Progression free survival
PMD: Progression metabolic disease
PMR: Partial metabolic response
PR: Partial response
RECIST: Response evaluation criteria in solid tumors
ROC: Receiver operating characteristic
SD: stable disease
SMD: Stable metabolic disease
SUL: SUV normalized to lean body mass
SUVmax: Maximum standardized uptake value
SUVmean: Mean standardized uptake value
